# CMCS: contrastive-metric learning via vector-level sampling and augmentation for code search

**DOI:** 10.1038/s41598-024-64205-2

**Published:** 2024-06-24

**Authors:** Qihong Song, Haize Hu, Tebo Dai

**Affiliations:** 1 School of Computer Science and Engineering, Hunan University of Science and Technology, Xiangtan, China; 2 Key Lab. of Knowledge Processing and Networked Manufacturing, Hunan University of Science and Technology, Xiangtan, Hunan China

**Keywords:** Code search, Multimodal contrastive learning, Metric learning, Hard negative sample, Engineering, Mathematics and computing

## Abstract

Code search aims to search for code snippets from large codebase that are semantically related to natural query statements. Deep learning is a valuable method for solving code search tasks in which the quality of training data directly impacts the performance of deep-learning models. However, most existing deep-learning models for code search research have overlooked the critical role of training data within batches, particularly hard negative samples, in optimizing model parameters. In this paper, we propose contrastive-metric learning CMCS for code search based on vector-level sampling and augmentation. Specifically, we propose a sampling method to obtain hard negative samples based on the K-means algorithm and a hardness-controllable sample augmentation method to obtain positive and hard negative samples based on vector-level augmentation techniques. We then design an optimization objective composed of metric learning and multimodal contrastive learning using obtained positive and hard negative samples. Extensive experiments were conducted on the large-scale dataset CodeSearchNet using seven advanced code search models. The results show that our proposed method significantly enhances the training efficiency and search performance of code search models, which is conducive to promoting software engineering development.

## Introduction

**Figure 1:**
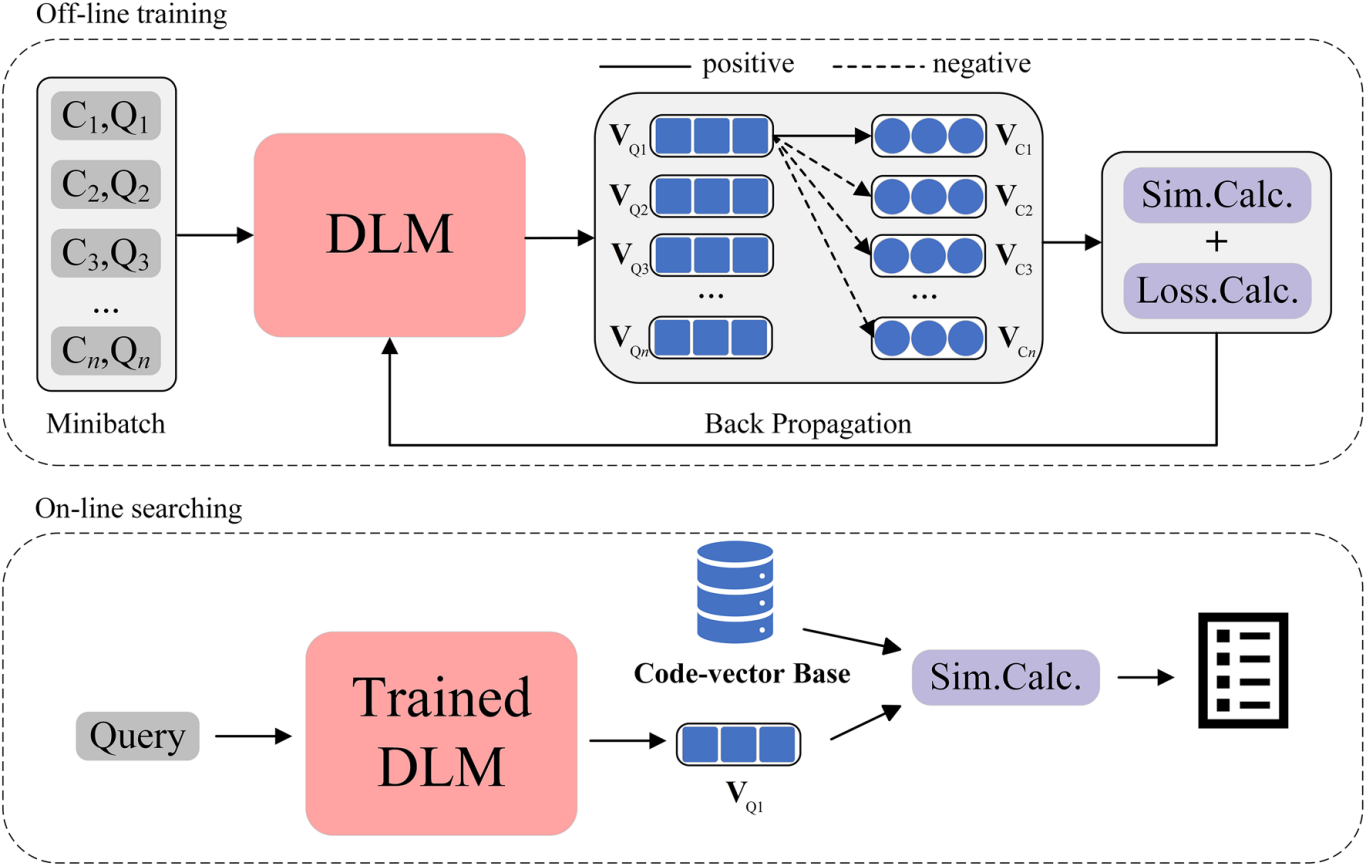
A general code search process based on deep learning.

Code search, retrieving the correct code snippets (positive code samples) from a large codebase according to a query statement^1^, is an essential part of software development. It can improve the efficiency of programmers coding by reusing and source code management. In early research, code search mainly relied on information retrieval techniques^2–4^ based on text similarity. However, this approach was limited by the gap between programming languages and natural languages in terms of syntax and expression^5^. With the development of deep learning, many deep learning models (DLM) for code search are proposed. They can align code snippets and query statements in high-dimensional vector space, thus reducing the semantic gap^6–8^. The general process is shown in Fig. 1. The process can be divided into off-line training and online search. Existing deep learning models are mainly based on metric learning^9^ as described in the off-line training part of Fig. 1, which reduces the distance between the given query (anchor query sample) and relevant code snippets (positive code samples) while increasing the distance between the anchor query sample and irrelevant code (negative code samples) in vector space.

**Figure 2:**
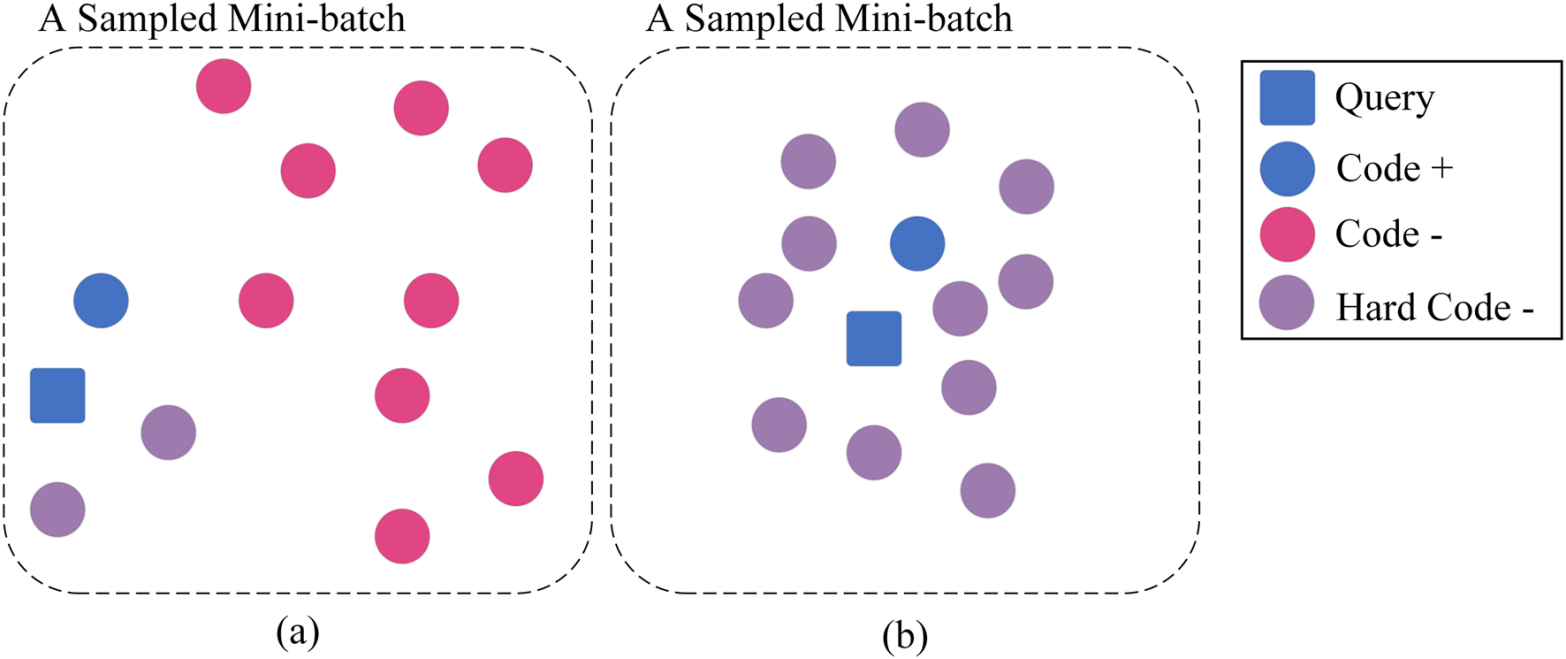
(**a**) Random sampling. (**b**) Sampling with hard negative samples.

Among the negative samples, there exist special samples that are close to the anchor sample but should be far away, i.e., they are similar to the anchor sample vector but have different semantics. The kind of negative samples are defined as hard negative samples. Hard negative samples can help guide a model to correct its mistakes more quickly and effectively during training, thus improving the performance of the model^10–15^. However, most existing code search models have not paid enough attention to the indispensable role of hard negative samples. Specifically, the training data of a minibatch is randomly sampled from the training set and the minibatch is composed in the form of related code-query pairs. Choose a query from the minibatch as an anchor sample, which corresponds to the only positive code sample in the minibatch. Most existing research considers the other code samples in this minibatch as negative code samples^16–18^. Since the minibatch is obtained through random sampling, only a few hard negative code samples exist within the negative code samples, which resulted in the negative samples being easily distinguishable from the anchor sample and thus cannot fully optimize the model. The distribution of the sample vectors within a randomly sampled batch versus the distribution of the sample vectors within a batch sampling hard negative samples is shown in Fig. 2.

Recently, some studies have introduced contrastive learning into code search^19–21^. Contrastive learning utilizes augmentation techniques to generate code snippets similar to positive samples and trains the model to distinguish the positive samples from the negative samples. In this way, contrastive learning improves the model's ability to capture the critical features of the samples. However, current research on contrastive learning for code search does not fully utilize the multimodal features of code snippets. Most focus solely on the semantic features of tokenized code sequences and syntactic features of code’s Abstract Syntax Tree (AST) while overlooking aspects such as control flow features and data flow features^22–24^. Augmentation techniques are essential to the unsupervised training of contrastive learning, and existing augmentation methods for code are generally divided into text-level and vector-level methods. Text-level augmentation generates corresponding positive samples by rewriting the original code sample^25,26^, including variable renaming, inserting meaningless statements, reordering independent statements, etc. Vector-level augmentation generates corresponding positive samples by perturbing the representation vector of the anchor code sample, which includes methods such as linear interpolation and stochastic perturbation^27^. However, most existing contrastive learning methods still focus on text-level augmentation. Contrastive learning aims to obtain augmented positive sample vectors and train the model with negative sample vectors. Positive samples generated through text-level augmentation need to be represented as vectors by the deep learning model. In contrast, vector-level augmentation directly generates positive sample vectors from the anchor sample vectors without representation. Therefore, text-level augmentation-based contrastive learning consumes more time and computational resources. Additionally, the negative samples in the minibatch for contrastive learning training are also randomly sampled as metric learning mentioned above, which also does not consider the beneficial influence of hard negative samples. Nevertheless, the augmentation techniques used for generating positive samples have inspired our idea of generating vector-level hard negative samples, which helps to overcome the limitation of relying solely on sampling to obtain hard negative samples.

To combine the benefits of metric and contrastive learning, and to fully leverage the hard negative samples and multimodal features of code, we propose an effective contrastive-metric learning for code search (CMCS) based on vector-level sampling and augmentation. CMCS can improve the training efficiency and effectiveness of code search models, which is achieved mainly through the following two key components. (1) Vector-level sampling and augmentation. Firstly, we fine-tune a pre-trained model based on metric learning and represent code snippets in the training set as vectors by integrating multimodal features. Secondly, we use the K-means algorithm to cluster these code vectors. Finally, based on the clustering results, we select code snippets of the anchor code sample’s same category as hard negative samples according to vector similarity. For sample augmentation, we propose a vector-level sample augmentation method that can control the hardness, which is used to generate hard negative samples and positive samples. (2) Contrastive-metric learning. Based on the hard negative and positive samples obtained by sampling and augmentation, we propose contrastive-metric learning for code search, combining metric and multimodal contrastive learning. Since code and query belong to two different languages, we treat them as two modalities. In order to fully utilize the various features of the code, we further parse the code into tokens, AST, Control Flow Graphs (CFG), and Data Flow Graphs (DFG). Based on the parsing results, they are serialized and represented as the semantic, syntactic, control, and data flow features of the code, respectively. These features are regarded as different modal features of the code. Finally, we use contrastive learning to train the model for each modality features using hard negative and positive samples. This approach facilitates the model in better understanding and representing code and query. Meanwhile, we also train the model using metric learning with the obtained hard negative samples, it helps the model better learn the association for relevant code-query pairs.

The contributions of this work can be summarized as follows:

- We propose a vector-level sampling method by K-means for hard negative samples, and a hardness-controllable vector-level augmentation method for positive and hard negative samples. For the augmentation method, we propose a fine-grained random augmentation strategy to increase the diversity of feature patterns of the obtained samples and reduce the impact of overfitting.
- Based on the sampling and augmentation method, we propose effective contrastive-metric learning for code search (CMCS). The contrastive learning part of this approach fully utilizes various features of the code and considers multiple modalities of input data, enabling the model to deeply learn the features of both the code and queries. The metric learning part enhances the model's ability to match relevant codes and queries.
- We train CMCS on six programming languages separately with seven state-of-the-art deep code models, and the results demonstrate that CMCS can effectively improve the training efficiency and search performance of the models.

The rest of the paper is organized as follows. Section "Methodology" introduces the details of CMCS, including sampling and augmentation methods and contrastive-metric learning. Section "Experimental evaluation" presents experimental evaluation of CMCS's performance. Section "Related work" introduces related work including code search and data augmentation. In section "Conclusions", we conclude the paper and propose our future work.

## Methodology

**Figure 3:**
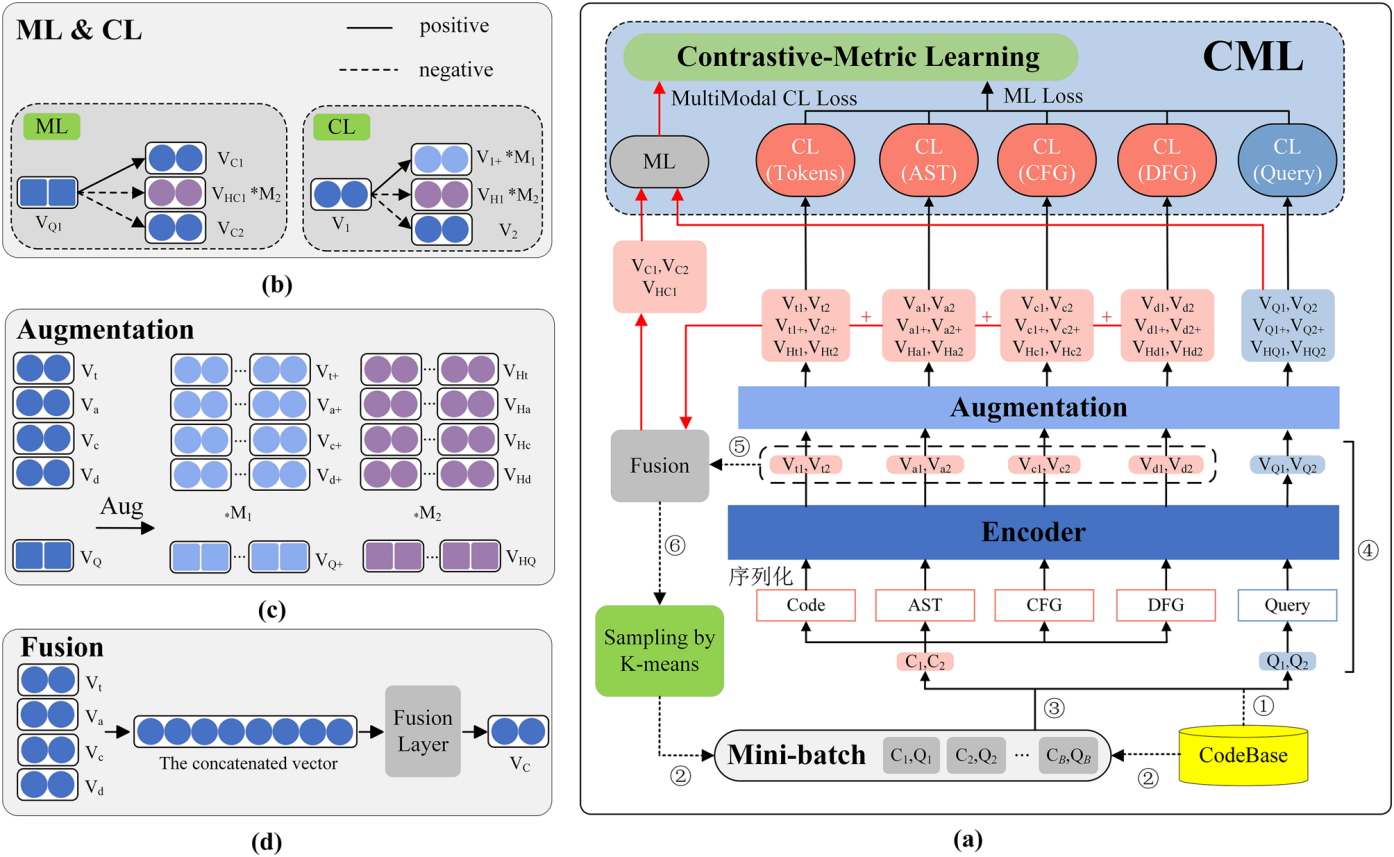
Overall architecture of CMCS. (**a**) Overall process of CMCS. (**b**) Details of the CL and ML parts. (**c**) Details of the augmentation part. (**d**) Details of the fusion part.

The overall architecture of CMCS is shown in Fig. 3a, which consists of the following four main components.

1. **The sampling method for hard negative samples** uses the K-means algorithm to cluster the pre-represented code vectors and perform sampling based on the clustering results. The code vectors used for clustering are obtained by fusing the multimodal features of the code represented by the encoder and fusion module.
2. **The encoder** separately represents the multimodal features of the inputs, including semantic features of the query, code semantic features, code syntactic features, code control flow features, and code data flow features.
3. **The hardness-controllable sample augmentation method** based on vector-level augmentation generates positive and hard negative samples for contrastive-metric learning.
4. **Contrastive-metric learning** (CML) consists of metric learning (ML) and multiple contrastive learning (CL) for different modal features, which jointly optimize the model and improve code search performance.

The details of the sampling method, multimodal representation, augmentation method, and contrastive-metric learning are elaborated as follows.

### The sampling method for hard negative samples

In this section, we introduce how to sample hard negative samples from the training set, which allows us to construct a mini-batch rich in hard negative samples. The sampling method is based on the vectors of code snippets. In the sampling before each epoch, we use the encoder and fusion module updated after the last epoch to obtain vectors that fuse the multimodal code features. The specific multimodal representation and fusion details will be expounded in sections "The multimodal representations of the input" and "Metric learning for CMCS". Before the first epoch of training, we use the unoptimized encoder and fusion module to represent the code multi-features based on metric learning, where the loss function fine-tuned by metric learning is as shown in Eq. (1):

1 $$ ML_{{{\text{Q}}i}} = - {\text{log}}\frac{{{\text{exp(}}{\mathbf{V}}_{{{\text{Q}}i}} \cdot {\mathbf{V}}_{{{\text{C}}i}} {)}}}{{{\text{exp(}}{\mathbf{V}}_{{{\text{Q}}i}} \cdot {\mathbf{V}}_{{{\text{C}}i}} {)} + \sum\nolimits_{j \ne i}^{B} {{\text{exp(}}{\mathbf{V}}_{{{\text{Q}}i}} \cdot {\mathbf{V}}_{{{\text{C}}j}} {)}} }} $$

where **V**_Q*i*_ represents an anchor query sample vector in a minibatch of size *B*, it generally has a corresponding semantically related code snippet vector **V**_C*i*_ as a positive sample. All other code snippet vectors **V**_C*j*_ (*i* ≠ *j*) in the batch are negative samples.

We specifically obtained the vectors of all code snippets in training set through steps ①④⑤⑥ shown in Fig. 3a. Subsequently, we use the K-means algorithm to cluster the code snippets into *K* clusters based on these vectors. The code snippets within the same cluster have a relatively close vector distance, as this algorithm classifies through vector distances. Since the functions of code snippets in the dataset are mutually exclusive, code snippets within the same cluster have different semantics but similar vectors, i.e., they are hard negative samples of each other. When constructing the mini-batch, we need to ensure that there are a certain number of hard negative samples in the minibatch to improve the training effect. Therefore, we only need to sample code samples equivalent to half of the batch size from the same cluster category, as they are hard negative samples for each other. To ensure the randomness and diversity of samples in the minibatch and to avoid overfitting, we randomly sample a number of negative samples from codebase equal to half of the batch size, which together with the sampled hard negative samples form a complete mini-batch, as shown in step ② of Fig. 3a.

### The multimodal representations of the input

Deep learning-based code search explores the relationship between code and query in vector space, so the model's ability to capture and represent the features of code and query determines the performance of code search. Many current studies, especially those involving contrastive learning in code search, only represent the token sequence of code snippets, with the vector representing the overall features of the code snippets. However, code has more than just semantic features represented by token sequences. Processed code can present multi-dimensional features, such as syntactic, data flow, and control flow features. These various features describe code snippets from different dimensions. Since code and query are two types of languages, we regard them as two modalities. In addition to representing the query modality, CMCS refines the code modality, mining various modality features of the input code and representing them separately. Based on multimodal representation vectors, CMCS uses metric contrastive learning to enable the model to perceive, learn, and represent more comprehensive and accurate code features, thereby improving the matching accuracy of relevant code and queries. The steps ③ and ④ in Fig. 3a describe the multimodal representation process of the input. For the sake of description, we assume that the batch size is 2, i.e., we operate on (C_1_, Q_1_) and (C_2_, Q_2_). For the multimodal representation of code, we first parse the code into code text segments, ASTs, CFGs, and DFGs, as shown in step ④ of Fig. 3a. Then, we convert them into sequences. Specifically, the code segment is tokenized to obtain token sequence, AST is pre-order traversed to obtain tree sequence, and CFG and DFG are separately processed to extract edge information, resulting in control flow and data flow sequences. Finally, the obtained sequences are represented as vectors **V**_t_, **V**_a_, **V**_c_, and **V**_d_ through the encoder, representing the semantic, syntactic, control flow, and data flow features of the code snippet, respectively. For the query, we only parse it into a token sequence for representation. As a result, we obtain the query modality feature **V**_Q_, and the above four code modality features **V**_t_, **V**_a_, **V**_c_, and **V**_d_.

### The hardness-controllable sample augmentation method

In this section, we first introduce the four vector-level augmentation methods used in CMCS. Then, we explain how to obtain the anchor samples' positive samples and hard negative samples by controlling the augmentation hardness. Finally, we discuss the strategies adopted in the CMCS augmentation process.

#### Vector-level augmentation method

The essence of the vector-level augmentation method is to generate new sample vectors based on the anchor vector by perturbing the vector features. Existing vector-level augmentation methods mainly include linear interpolation, stochastic perturbation, binary interpolation, and Gaussian scaling.

(1) Linear interpolation^28^.

Linear interpolation mainly uses the features of another sample to augment the anchor sample, and the method is calculated as Eq. (2).

2 $$ {\mathbf{V}}_{i}^{*} = \lambda {\mathbf{V}}_{i} + (1 - \lambda ){\mathbf{V}}_{j} $$

where **V**_*i*_ is the anchor sample vector, **V**_*j*_ is randomly sampled from other samples. *λ* is the interpolation coefficient sampled from a uniform distribution *U* (*α*, *β*), and *α*, *β* are mutable parameters near 1.0.

(2) Stochastic perturbation^29^.

Stochastic perturbation randomly deactivates some features of the sample to obtain a new sample, and the method is shown as Eq. (3).

3 $$ {\mathbf{Vf}}_{i}^{*} (e) = \xi {\mathbf{Vf}}_{i} (e) $$

where **Vf**_*i*_ (*e*) represents the *e*-th dimension feature of the sample vector **V**_*i*_, assuming that *ξ* is sampled from a Bernoulli distribution *B* (*e*, *ρ*) to control whether the feature is deactivated. *ρ* is a small deactivating probability value. In implementation, the Dropout layer is generally used for stochastic perturbation.

(3) Binary interpolation^30^.

Binary interpolation randomly swaps the feature **Vf**_*i*_ (*e*) of the anchor sample vector with the feature **Vf**_*j*_ (*e*) of another sample to generate a new sample vector as Eq. (4).

4 $$ {\mathbf{Vf}}_{i}^{*} (e) = \xi ({\mathbf{Vf}}_{i} (e) + {\mathbf{Vf}}_{j} (e)) + (1 - \xi ){\mathbf{Vf}}_{i} (e) $$

where *ξ* ~ *B* (*e*, *ρ*), control whether the feature is chosen to swap. *ρ* is a small deactivating (swapping) probability value.

(4) Gaussian scaling^31^.

Gaussian scaling augments the sample vector **V**_*i*_ by scaling it by a small factor, which can be viewed as adding perturbation noise to the sample as Eq. (5).

5 $$ {\mathbf{V}}_{i}^{*} = \mu {\mathbf{V}}_{i} $$

where *μ* is the scaling coefficient sampled from a Gaussian distribution *N* (0, *σ*) with small values of *σ*.

#### Hardness-controllable augmentation

Regarding the positive and negative samples and contrastive learning, we have two observations: (1) In the vector space, the essential difference between positive and negative samples of an anchor sample is the vector distance. The vector distance between a positive sample and an anchor sample is small. The vector distance between a negative sample and an anchor sample is far. In fact, the difference between positive and negative samples, including hard negative samples, is mainly manifested in the vector distance from the anchor sample. (2) In contrastive learning, using data augmentation techniques to generate positive samples essentially means generating samples similar to the anchor samples. Vector-level augmentation techniques directly perturb the representation vectors to generate vectors with high vector similarity to the anchor vectors as positive samples.

Inspired by the above two observations, we use vector-level augmentation techniques to generate representation vectors with different similarities (hardness) to the anchor samples as positive and hard negative samples. To the best of our knowledge, we are the first to propose vector-level augmentation for hard negative samples.

Specifically, the four augmentation methods are all essentially based on perturbing the vector. Among them, the *λ* (controlled by *α* and *β*) of linear interpolation, *ρ* of stochastic perturbation, *ξ* (controlled by *ρ*) of binary interpolation, *μ* (controlled by *σ*) of Gaussian scaling all control the degree of perturbation, which determines the similarity (hardness) between the augmented samples and the anchor samples. The four augmentation methods can be abstracted by the perturbation coefficients as an augmentation function, as shown in Eq. (6):

6 $$ {\mathbf{V}}_{i}^{*} (\theta ) = \theta {\mathbf{V}}_{i} (e) + \xi (1 - \theta ){\mathbf{V}}_{j} (e) $$

where *ξ* ~ *B* (*e*, *ρ*) controls whether to import another sample, and *θ* ∈ (0, 1.0] represents hardness. The larger the hardness, the more similar the generated samples are to the anchor samples, which can be used as positive samples for contrastive learning. The smaller the hardness, the greater difference between the generated samples and the anchor sample, and they are considered as negative samples. Hard negative samples refer to the appropriate range of hardness value between the hardness value of positive and negative samples, and the optimal hardness value will be discussed in the experimental section.

Therefore, we augment positive and hard negative samples by controlling the hardness of the augmentation. Our proposed CMCS samples the pairs of samples (C_1_, Q_1_), (C_2_, Q_2_)…(C_*B*_, Q_*B*_) to assemble the minibatch of size *B*. For a given sample pair (C_*i*_, Q_*i*_), we first parse the code C_*i*_ into a token sequence, an AST sequence, a CFG sequence, and a DFG sequence, and parse the query Q_*i*_ into a token sequence. Then, the encoder represents these five sequences to obtain the corresponding five modal feature vectors **V**_t*i*_, **V**_a*i*_, **V**_c*i*_, **V**_d*i*_ and **V**_Q*i*_. For each modal feature vector, one of the four augmentation methods is randomly selected and used to augment the vector *M1* and *M2* times with randomly select from a certain range of perturbations under different hardness values to obtain *M1* positive sample vectors **V**_t*i*+_, **V**_a*i*+_, **V**_c*i*+_, **V**_d*i*+_ and **V**_Q*i*+_ and *M2* hard negative sample vectors **V**_Ht*i*_, **V**_Ha*i*_, **V**_Hc*i*_, **V**_Hd*i*_ and **V**_HQ*i*_. Intuitively, as shown in the Fig. 3c, the dark blue circles and squares represent the original modal feature vectors. The light blue circles and squares represent the positive modal feature vectors obtained by augmenting the original vectors, and the purple circles and squares represent the hard negative modal feature vectors obtained by augmenting the original vectors.

#### Fine-grained random augmentation strategy

We have four augmentation methods as shown in Eqs. (2)–(5). Each method generates positive and hard negative sample vectors from a given anchor sample vector by controlling the perturbation coefficient according to different hardness value. The perturbation coefficient determines the degree of perturbation applied to the anchor sample vector by the augmentation method. The hardness mentioned in this paper abstracts the perturbation coefficient across all augmentation methods, represented as *θ* of Eq. (6).

The fine-grained random augmentation strategy refers to randomly applying different augmentation methods and randomly determining varying perturbation amounts under a given perturbation coefficient for each sample vector. This approach ensures the richness and diversity of the generated samples, reducing the likelihood of overfitting and enhancing the model's training performance. The fine-granularity of this strategy is manifested in the randomness it introduces in the perturbation magnitude under the corresponding perturbation coefficients assigned to different augmentation methods. In CMCS, we need to generate multiple positive sample vectors and hard negative sample vectors for the query semantic feature vector **V**_Q_ and the multimodal feature vectors of the code, including the code semantic feature vector **V**_t*i*_, the code syntax feature vector **V**_a*i*_, the code control flow feature vector **V**_c*i*_, and the code data flow feature vector **V**_d*i*_. The strategy introduced in this section involves randomly selecting one of the methods for each augmentation of different given sample vectors. It's important to note that the given perturbation coefficients only set the range for the perturbation's degree of the corresponding augmentation method. The specific perturbation amount within this range is variable. Therefore, the augmentation strategy proposed in this section not only randomly selects the augmentation method, but also randomly selects the perturbation amount within the range determined by the corresponding perturbation coefficients. This fine-grained random perturbation ensures the diversity of the generated sample's feature patterns.

### Contrastive-metric learning

Based on the samples obtained through sampling and augmentation, this section will separately introduce metric learning, multimodal contrastive learning, and the overall process of contrastive-metric learning.

#### Multimodal contrastive learning for CMCS

Contrastive learning learns the features of the samples unsupervised by distinguishing the positive samples generated by the augmentation techniques. Code and query belong to the programming language and natural language, which have significant semantic and syntactic differences and can be considered two modalities. CMCS also parses code snippets into token sequences, ASTs, CFGs, and DFGs. Therefore, the modality of the code is further subdivided into semantic, syntactic, control flow, and data flow feature modalities. In this section, we use the positive and hard negative samples obtained through sampling and augmentation to perform contrastive learning separately on the query modality and four code modalities, thereby improving the model's ability to extract features from code and query.

After the samples in the mini-batch are parsed, serialized, and represented, we obtain five types of code modality feature vectors {**V**_t*i*_, **V**_a*i*_,**V**_c*i*_,**V**_d*i*_}*B i* = 1 and query modality feature vectors {**V**_Q*i*_}*B i* = 1. CMCS performs contrastive learning for each modality feature individually, allowing the model to learn the features of each modality. Specifically, let **V**_*i*_ represent one type of modality feature vector of the *i*th sample pair (C_*i*_, Q_*i*_) in the mini-batch, and we perform augmentations to generate the corresponding positive sample set {{**V***m i* +}*M1* m = 1} *B i* = 1 and hard negative sample set {{**V***m* H*i*}*M2* m = 1} *B i* = 1. As known from Section "The sampling method for hard negative samples", each mini-batch is composed of sampled hard negative samples and randomly sampled general negative samples. Therefore, in the mini-batch, other samples of the same modality feature **V**_*k*_ (*k* ≠ *i*) are not only negative samples for **V**_*i*_, but also contain a certain proportion of hard negative samples for **V**_*i*_. In this way, in the contrastive learning training of each modality feature, there is a certain proportion of hard negative samples in the negative samples, which is more conducive to the effect of contrastive learning. The contrastive learning loss function for a certain modality feature is shown in Eq. (7):

7 $$ L_{i}^{{\text{C}}} = - {\text{log}}\frac{{\sum\nolimits_{m = 1}^{M1} {{\text{exp(}}{\mathbf{V}}_{i} \cdot {\mathbf{V}}_{i + }^{m} {)}} }}{{\sum\nolimits_{m = 1}^{M1} {{\text{exp(}}{\mathbf{V}}_{i} \cdot {\mathbf{V}}_{i + }^{m} {)}} + \sum\nolimits_{k = 1}^{M2} {{\text{exp(}}{\mathbf{V}}_{i} \cdot {\mathbf{V}}_{{{\text{H}}i}}^{k} {)} + \sum\nolimits_{j = 1,j \ne i}^{B} {{\text{exp(}}{\mathbf{V}}_{i} \cdot {\mathbf{V}}_{j} {)}} } }} $$

Therefore, we can obtain the contrastive learning loss function *L*C t*i* for query semantic modality features, *L*C a*i* for code semantic modality features, *L*C c*i* for code syntactic modality features, *L*C d*i* for code control flow modality features, and *L*C Q*i* for code data flow modality features.

Intuitively, as shown in the CL part of Fig. 3b, the blue circles on the left represent a modality feature vector **V**_1_, and the light blue circles on the right represent the generated *M1* positive modality feature vectors {**V***m* 1 +}*M1 m* = 1, the purple circles represent the generated *M2* hard negative modality feature vectors {**V***m H1* +}*M2 m* = 1, and the dark blue circles on the right represent the negative modality feature vectors **V**_2_ from mini-batch. Each modality feature vector has *M1* semantically related positive modality feature vectors connected by the solid line. The other vectors are all the negative samples connected by the dashed line.

In summary, we can calculate the contrastive learning loss values for the multimodal features of code, as well as for the query modal features. This multimodal contrastive learning approach improves the model's ability to understand and represent the features of code and query.

#### Metric learning for CMCS

In a minibatch of size *B*, the four types of original modal feature vectors {**V**_t*i*_, **V**_a*i*_, **V**_c*i*_, **V**_d*i*_}*B i* = 1 and query vectors {**V**_Q*i*_}*B i* = 1 obtained through parsing, serialization, and representation are augmented *M*2 times to generate corresponding hard negative samples {{**V***m* Ht*i*}*M2 m* = 1, {**V***m* Ha*i*}*M2 m* = 1, {**V***m* Hc*i*}*M2 m* = 1, {**V***m* Hd*i*}*M2 m* = 1, {**V***m* HQ*i*}*M2 m* = 1} *B i* = 1.

Next, as shown in Fig. 3d, we use a fusion module composed of a fully connected neural network to fuse the four modal feature vectors **V**_t*i*_, **V**_a*i*_, **V**_c*i*_ and **V**_d*i*_, as well as the augmented hard negative modal feature vectors **V***m* Ht*i*, **V***m* Ha*i*, **V***m* Hc*i* and **V***m* Hd*i* of the same code sample C_*i*_. Eventually, we obtain the complete original code vector **V**_C*i*_ and the augmented hard negative code vector **V***m* HC*i*, both of which integrate all modal features. If *Fusion* denotes the fusion operation of the neural network, then the operation processes can be represented as Eqs. (8) and (9).

8 $$ {\mathbf{V}}_{{{\text{C}}i}} = Fusion{(}{\mathbf{V}}_{{{\text{t}}i}} ,{\mathbf{V}}_{{{\text{a}}i}} ,{\mathbf{V}}_{{{\text{c}}i}} ,{\mathbf{V}}_{{{\text{d}}i}} {)} $$

9 $$ {\mathbf{V}}_{{{\text{HC}}i}}^{m} = Fusion{(}{\mathbf{V}}_{{{\text{Ht}}i}}^{m} ,{\mathbf{V}}_{{{\text{Ha}}i}}^{m} ,{\mathbf{V}}_{{{\text{Hc}}i}}^{m} ,{\mathbf{V}}_{{{\text{Hd}}i}}^{m} {)} $$

For a sample pair (C_*i*_, Q_*i*_) in the minibatch, we obtain its representation vectors (**V**_C*i*_, **V**_Q*i*_) and the hard negative sample vectors {**V*m* HC*i***, **V*m* HQ*i***}*M2 m* = 1 by augmentation. Metric learning aims to reduce the vector distance between related code query pairs and increase the distance between unrelated code query pairs. For a query, its unrelated code samples include not only the unrelated code samples in the mini-batch, but also the corresponding hard negative code samples generated by the augmentation method. Specifically, for the *i*th query vector **V**_Q*i*_, its semantically related code vector is **V**_C*i*_, and the semantically unrelated samples are {**V*m* HC*i***}* M2 m* = 1 and **V**_C*j*_ (*i* ≠ *j*). We use dot product to calculate the vector similarity. The metric learning loss value of the query **V**_Q*i*_ can be calculated as Eq. (10).

10 $$ L_{i}^{{\text{M}}} = - {\text{log}}\frac{{{\text{exp(}}{\mathbf{V}}_{{{\text{Q}}i}} \cdot {\mathbf{V}}_{{{\text{C}}i}} {)}}}{{{\text{exp(}}{\mathbf{V}}_{{{\text{Q}}i}} \cdot {\mathbf{V}}_{{{\text{C}}i}} {)} + \sum\nolimits_{k = 1}^{M2} {{\text{exp(}}{\mathbf{V}}_{{{\text{Q}}i}} \cdot {\mathbf{V}}_{{{\text{HC}}k}} {) + }\sum\nolimits_{j = 1,j \ne i}^{B} {{\text{exp(}}{\mathbf{V}}_{{{\text{Q}}j}} \cdot {\mathbf{V}}_{Ci} {)}} } }} $$

Intuitively, as shown in the ML part of Fig. 3b, the dark blue square on the left represents a certain query sample vector. The dark blue circles in the first row on the right represent the related code sample vectors of the query, and the purple circles in the second row represent the augmented *M2* corresponding hard negative code sample vectors. The remaining dark blue circles represent other code sample vectors in the minibatch serve as negative samples.

#### Contrastive-metric learning

The previous two sections proposed corresponding loss functions from the perspectives of metric learning and multimodal contrastive learning, respectively. In order to combine the advantages of contrastive learning and metric learning, we integrate metric learning and multimodal contrastive learning to train the model's feature representation ability and its ability to match relevant code query pairs. For an minibatch of size *B*, the total contrastive-metric learning loss is obtained based on Eq. (11):

11 $$ L = \sum\nolimits_{i = 1}^{B} {{(}L_{{{\text{Q}}i}}^{{\text{M}}} + L_{{{\text{Q}}i}}^{{\text{C}}} + L_{{{\text{t}}i}}^{{\text{C}}} + L_{{{\text{a}}i}}^{{\text{C}}} + L_{{{\text{c}}i}}^{{\text{C}}} + L_{{{\text{d}}i}}^{{\text{C}}} {)}} $$

where *L* represents the total loss of a minibatch. *L*M Q*i* represents the metric learning loss, *L*C Q*i* represents the query semantic modality contrastive learning loss, and *L*C t*i*, *L*C a*i*, *L*C c*i*, and *L*C d*i* separately represent the code semantic, syntax, control flow, and data flow modality contrastive learning losses. The pseudocode for our proposed Contrastive-metric learning is shown below.

**Algorithm 1:**
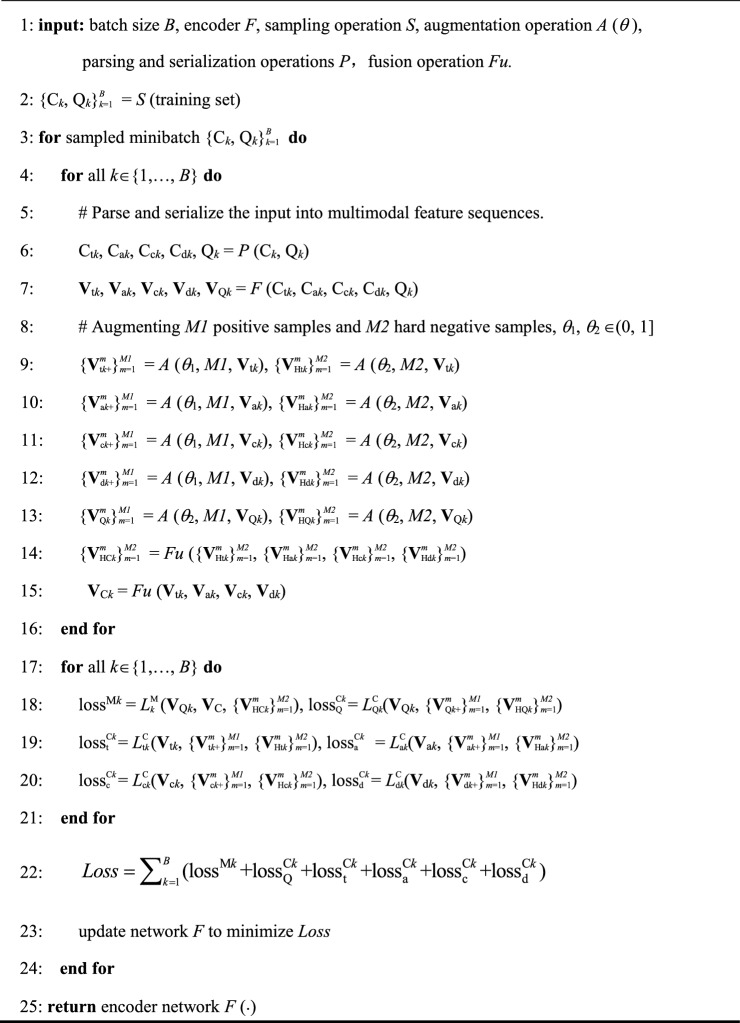
Contrastive-metric learning algorithm.

## Experimental evaluation

In order to investigate the structural rationality and the performance of CMCS on code search, extensive experiments are conducted to answer the following research questions:

**RQ1:** How effective is CMCS in code search task?

**RQ2:** How is the training efficiency of CMCS?

**RQ3:** How do the different components of CMCS affect the model's performance?

**RQ4:** Is it possible for the CMCS to overfit? How should this risk be reduced?

**RQ5:** How to choose the optimal parameters to ensure high performance of CMCS?

### Experimental setup

#### Dataset

In this work, we use CodeSearchNet^32^ large-scale dataset to evaluate the effectiveness of the proposed model. The dataset contains six different programming language data stored as semantically related code-query pairs. We filtered the dataset following the method of Guo et al.^33^, removing low-quality data (such as code that cannot be parsed into abstract syntax trees, code that is too long or short, or contains special characters). We also extracted a large number of code snippets according to the programming language type from the entire corpus to form the search codebase used for validation and test. The details of the preprocessed dataset are shown in Table 1.

**Table 1:** The details of the filtered CodeSearchNet dataset.

Language	Training	Validation	Test	Search codebase
Python	251,820	13,914	14,918	43,827
PHP	241,241	12,982	14,014	52,660
Go	167,288	7325	8122	28,120
Java	164,923	5183	10,955	40,347
JavaScript	58,025	3885	3291	13,981
Ruby	24,927	1400	1261	4360

#### Baselines

This paper compares the proposed CMCS with seven advanced code search models. Three models (SyncoBERT, CodeRetriever and CoCoSoDa) use contrastive learning techniques, two models (MRCS and TabCS) utilize the multimodal properties of code, and two models (CodeBERT and GraphCodeBERT) are pre-trained models fine-tuned on code search tasks. The baseline models are presented as follows.

**MRCS**^34^ is is an advanced multimodal representation model proposed by Gu et al. The model proposes four tree-sequence representations generated based on traversal and sampling of the code. This paper uses Tokens + SBT, which has the best overall performance, as the multimodal input for code representation.

**TabCS**^35^ is a two-stage attention-based code recommendation model proposed by Xu et al.^17^. Through a two-stage attention mechanism, code and query are represented based on the correlation between the features of inputs.

**CodeBERT**^36^ is a bimodal pre-trained model for programming language and natural language. It is pre-trained using two tasks: masked language modeling (MLM) and replaced token detection.

**GraphCodeBERT**^33^ is a pre-trained model that incorporates code semantic structure information, and it has three pre-training tasks: MLM, code structure edges prediction, and alignment representations of source code and code structure.

**SyncoBERT**^21^ is multi-modal contrastive pre-training for code representation. It takes source code, abstract syntax tree (AST) and summarization as input and pretrained with identifier prediction and AST edge prediction.

**CodeRetriever**^19^ is a pre-training code model that learns the function-level code semantic representations through large-scale code-text contrastive pre-training.

**CoCoSoDa**^20^ is a code search model that effectively uses contrastive learning. It proposes soft data augmentation and momentum mechanism to enhance the effect of contrastive learning, which are used to generate positive and negative samples at the text level, respectively.

#### Evaluation metrics

To evaluate the effectiveness of CMCS, we utilize the three most widely used metrics for code search: Normalized Discounted Cumulative Gain (NDCG), Mean Reciprocal Rank (MRR), and SuccessRate@k (SR@k).

MRR^8^ measures the ranking of the target code in the returned code list, and only cares about the ranking of the most relevant code. The higher the MRR value, the higher the first hit code is ranked.

12 $$ MRR = \frac{1}{\left| Q \right|}\sum\limits_{j = 1}^{\left| Q \right|} {\frac{1}{{Rank_{j} }}} $$

where *Q* is the number of query in the valid/test set, Rank_*j*_ is the ranking position of the most relevant code in the returned list for the *j*th query.

NDCG^34^ measures the similarity between the code recommendation list returned by the model and the ideal code recommendation list, and it considers the overall ranking of the the returned code list. A higher NDCG value indicates better overall ranked results.

13 $$ NDCG = \frac{1}{\left| Q \right|}\sum\limits_{j = 1}^{k} {\frac{{2^{{r_{j} }} - 1}}{{\log_{2} {(}1 + j{)}}}} $$

where *Q* is the number of query in the valid/test set, r_*j*_ is the relevance of the code at position *j* in the returned top-*k* search results to the query, and *k* denotes the maximum value that NDCG can give the query.

SuccessRate@k^35^ measures the probability that the most relevant code is in the top-*k* of the returned code list. The higher the metric value, the higher the hit rate of the returned code list.

14 $$ Success\,\, Rate@k = \frac{1}{\left| Q \right|}\sum\limits_{j = 1}^{\left| Q \right|} {Hit{(}Q_{j} ,k{)}} $$

where *Q* is the number of query in the valid/test set, for the *j*th query statement Q_*j*_, *Hit* is a function that shows whether the most relevant code appears in the top-*k* code of the returned list.

#### Implementation details

All experiments were conducted in a Linux environment using Nvidia GTX 3090 24 GB. For the baseline model, we followed all experimental settings from the original paper except for the training epoch. For the proposed CMCS, the experimental details are as follows.

We input the model with all training data as code-query pairs during training. During validation/test, we randomly sample 1000 query statements from the validation/test set, search the codebase in the dataset for relevant code snippets, and return the top 50 code snippets for each query. We use CodeBERT as the encoder for CMCS. For sample sampling, we fine-tune the CodeBERT to represent code snippets based on Eq. (1) and cluster the code vectors into* K* = 32 clusters for hard negative sample sampling. For sample augmentation, we randomly choose one of the four augmentation methods for each sample, generating five positive samples and five hard negative samples for contrastive-metric learning. The hardness parameter *θ* for augmenting positive samples is set to 0.94, and the hardness parameter θ for augmenting hard negative samples is set to 0.70. We set the number of training epochs to 10, the batch size to 32, and the learning rate to 2e−5.

### RQ1: the effectiveness of CMCS.

In this section, we evaluate the superiority of CMCS in code search using MRR, NDCG, and SR@1. Specifically, we evaluate the performance of CMCS and seven baseline models on the code search task using six programming language datasets of CodeSearchNet. The results are shown in Table 2.

**Table 2:** Effectiveness of CMCS compared with the baseline models.

Model		Ruby	Python	Java	PHP	Go	JavaScript	Average
CMCS	MRR	**0.821**	**0.765**	**0.774**	**0.712**	**0.922**	**0.768**	**0.794**
	NDCG	**0.835**	**0.774**	**0.792**	0.724	**0.945**	**0.785**	**0.809**
	SR@1	**0.833**	**0.769**	**0.785**	0.719	0.933	**0.781**	**0.803**
TabCS	MRR	0.464	0.509	0.503	0.512	0.524	0.482	0.499
	NDCG	0.501	0.545	0.557	0.532	0.548	0.516	0.533
	SR@1	0.482	0.523	0.534	0.519	0.539	0.502	0.517
MRCS	MRR	0.470	0.576	0.632	0.564	0.661	0.500	0.567
	NDCG	0.511	0.599	0.651	0.589	0.682	0.544	0.596
	SR@1	0.493	0.582	0.644	0.577	0.678	0.523	0.583
CodeBERT	MRR	0.633	0.642	0.674	0.619	0.878	0.595	0.674
	NDCG	0.654	0.669	0.692	0.643	0.889	0.624	0.695
	SR@1	0.649	0.655	0.684	0.623	0.889	0.621	0.687
GraphCodeBERT	MRR	0.709	0.690	0.689	0.648	0.896	0.637	0.712
	NDCG	0.731	0.722	0.705	0.671	0.923	0.655	0.735
	SR@1	0.723	0.712	0.699	0.662	0.912	0.648	0.726
SyncoBERT	MRR	0.721	0.722	0.720	0.662	0.908	0.675	0.735
	NDCG	0.735	0.749	0.762	0.695	0.944	0.965	0.808
	SR@1	0.727	0.734	0.749	0.683	0.921	0.684	0.750
CodeRetriever	MRR	0.743	0.750	0.754	0.694	0.921	0.706	0.761
	NDCG	0.783	0.782	0.782	0.716	0.733	0.749	0.758
	SR@1	0.755	0.768	0.762	0.726	0.932	0.731	0.779
CoCoSoDa	MRR	0.802	0.759	0.761	0.704	0.920	0.750	0.783
	NDCG	0.825	0.772	0.783	**0.735**	0.942	0.779	0.806
	SR@1	0.813	0.764	0.774	**0.723**	**0.935**	0.771	0.797

Significant values are given in bold.

The experimental results show that the proposed CMCS significantly improved the three evaluation metric values of seven benchmark models on six programming languages. Specifically, it increased the MRR values of TabCS, MRCS, CodeBERT, GraphCodeBERT, SyncoBERT, CodeRetriever, and CoCoSoDa by 59.12%, 40.04%, 17.80%, 11.52%, 8.03%, 4.34%, and 1.40%, respectively.

**Figure 4:**
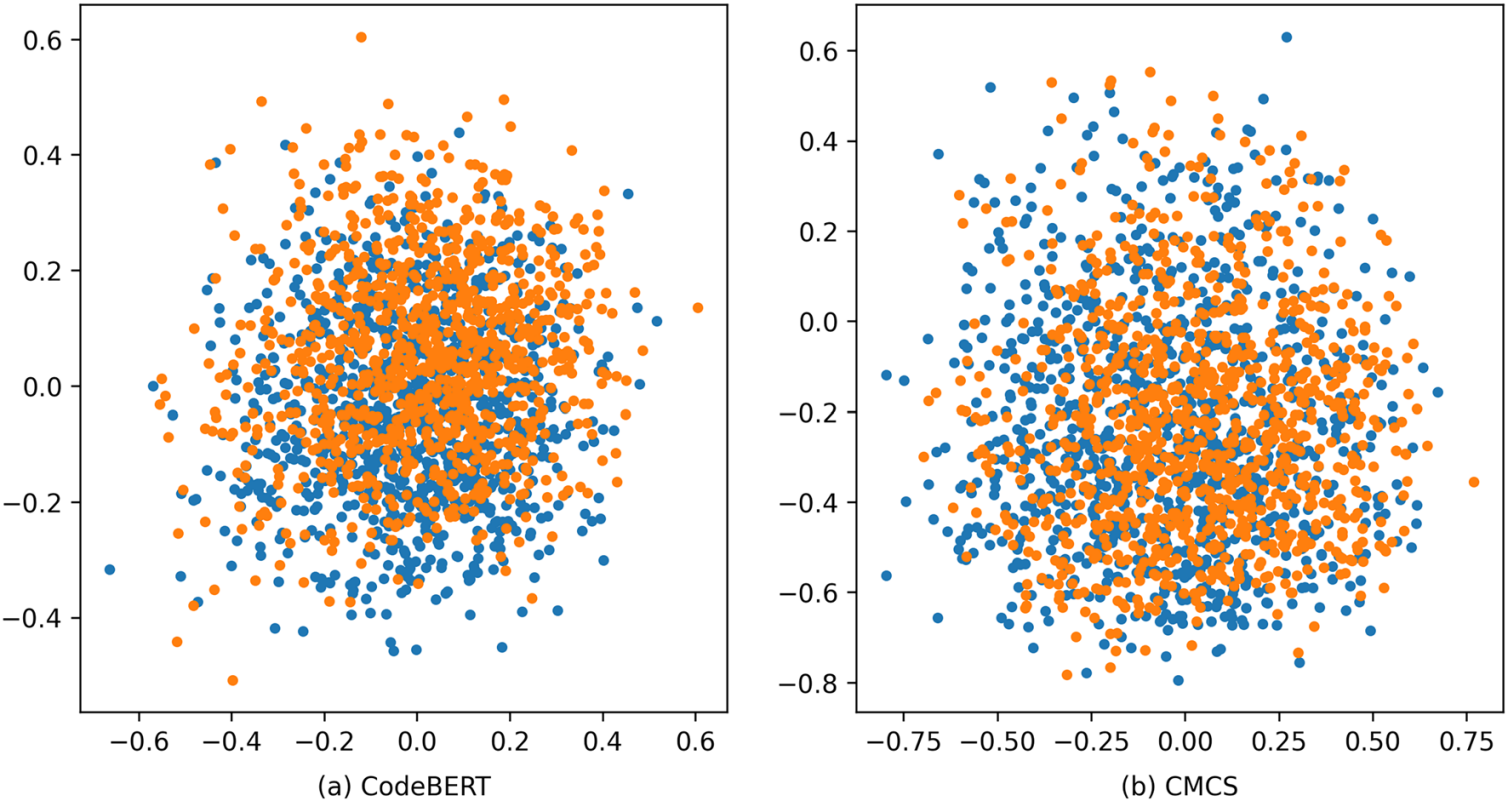
Visualization of code and query vectors distribution.

We mapped the distribution of 1000 code-query pairs’ vectors in two-dimensional space and plotted them in Fig. 4, where each yellow dot represents a code snippet, and each blue dot represents a query statement. Figure 4a represents the distribution of vectors after being represented by CodeBERT. We can see that most of the queries is densely clustered around the code, and there is difficulty in finding the relevant code for the query based on the shortest vector distance. Figure 4b represents the distribution of vectors after being represented by CMCS using the same code-query pairs. We can find that after training with CMCS, the distribution of code and query vectors is more dispersed and uniform, and it is much easier to find codes related to a query based on vector distance. Compared with Fig. 4a, the vector distances between points in Fig. 4b have been stretched, and the matching relationship between code and queries is more apparent, which is more beneficial to improve the performance of code search. The above experimental results and vector distribution all prove the positive role of CMCS in code search tasks.

### RQ2: the training efficiency of CMCS

Although large deep learning models have outstanding performance, they require substantial computing and time resources, so they generally take a long time to train. CMCS generates hard negative samples based on sampling and augmentation methods, which are beneficial for the model to correct errors more quickly during training and enable the model parameters to converge more rapidly. We use the total training time after the model has reached basic convergence to reflect the efficiency of training. The shorter the total training time, the higher the training efficiency of the model. We train each of the seven baseline models and the CMCS model on the Java dataset and record their training time (the unit is hour), and the results are shown in Table 3.

**Table 3:** Training efficiency of CMCS compared with baseline models.

Model	Convergence time (h)	Change ratio
CMCS	8.5	–
TabCS	8.1	↓4.7%
MRCS	10.2	↑20.0%
CodeBERT	11.8	↑38.8%
GraphCodeBERT	13.5	↑58.8%
SyncoBERT	15.6	↑83.5%
CodeRetriever	13.5	↑58.8%
CoCoSoDa	14.5	↑70.6%

The results in Table 3 show that the convergence time of the CMCS model is significantly reduced compared to the six baseline models, but it slightly increases compared to the TabCS model. Since TabCS only uses a simple neural network structure for training, the number of model parameters is greatly reduced compared to CMCS and the other six baseline models, so it has the shortest training time. However, its code search performance is the weakest. In order to sample hard negative samples, CMCS performs representation and clustering before each epoch of training, which consumes a certain amount of time. However, this time consumption is meaningful. Benefiting from the hard negative samples derived from sampling and augmentation, they help CMCS quickly learn the features of the code and the query, as well as the correlation between the code and the query, greatly shortening its training time. From the above analysis, we can conclude that CMCS can significantly improve training efficiency while ensuring code search performance.

### RQ3: rationality of CMCS’s architecture

In this section, we evaluate the rationality of the CMCS architecture, including the roles of main components in CMCS and the optimal usage strategy for the four augmentation methods.

#### Ablation experiment

We conducted ablation experiments on CMCS to evaluate the effects of metric learning (ML), contrastive learning (CL), multimodal contrastive learning (MCL), and the hard negative samples (Hard) on the code search performance using MRR, NDCG, and SR@1. The results are shown in Table 4. Experimental results show that removing any part of CMCS degrades the code search performance. It should be noted that the removal of MCL refers to the exclusive use of contrastive learning to study the semantic information modality of the code, and the removal of CL indicates the non-utilization of any contrastive learning module. The results indicate that the ML, MCL, and Hard components all play essential roles in the performance of CMCS, and the proposed architecture is relatively reasonable. We can draw the following conclusion: multimodal contrastive learning enables the model to learn more code and query features, metric learning enables the model to learn the matching relationship between code and query, and hard negative sampling benefits training performance.

**Table 4:** The ablation experiment results of CMCS.

Language		Ruby	Python	Java	PHP	Go	JavaScript	Average
CMCS	MRR	**0.821**	**0.765**	**0.774**	**0.712**	**0.922**	**0.768**	**0.794**
	NDCG	**0.835**	**0.774**	**0.792**	**0.724**	**0.945**	**0.785**	**0.809**
	SR@1	**0.833**	**0.769**	**0.785**	**0.719**	**0.933**	**0.781**	**0.803**
-ML	MRR	0.753	0.716	0.733	0.669	0.911	0.675	0.743
	NDCG	0.772	0.734	0.762	0.685	0.915	0.700	0.761
	SR@1	0.765	0.725	0.749	0.681	0.913	0.688	0.754
-CL	MRR	0.642	0.655	0.682	0.620	0.888	0.609	0.683
	NDCG	0.662	0.671	0.704	0.661	0.901	0.623	0.704
	SR@1	0.654	0.667	0.691	0.642	0.892	0.615	0.694
-MCL	MRR	0.693	0.681	0.693	0.638	0.898	0.624	0.705
	NDCG	0.723	0.724	0.729	0.654	0.724	0.642	0.699
	SR@1	0.712	0.694	0.711	0.649	0.911	0.635	0.719
-Hard	MRR	0.805	0.742	0.749	0.688	0.916	0.683	0.764
	NDCG	0.814	0.769	0.768	0.712	0.925	0.711	0.783
	SR@1	0.811	0.764	0.758	0.691	0.922	0.697	0.774

Significant values are given in bold.

#### The utilization strategy of augmentation methods

We have four augmentation methods: linear interpolation, stochastic perturbation, binary interpolation, and Gaussian scaling. We want to explore how to use the four augmentation methods to achieve optimal performance of CMCS. We conducted experiments on the CodeSearchNet to verify five expansion strategies using MRR: using a single method to augment samples (using the same expansion method each time), mixing all four methods to augment samples (randomly selecting one of the four methods each time), and the results are shown in Table 5.

**Table 5:** The results for augmentation strategy of CMCS.

Augmentation method	Ruby	Python	Java	PHP	Go	JavaScript
No augmentations	0.642	0.655	0.682	0.620	0.888	0.609
Linear interpolation	0.796	0.744	0.745	0.695	0.887	0.738
Stochastic perturbation	0.793	0.742	0.739	0.689	0.893	0.741
Binary interpolation	0.815	0.751	0.726	0.705	0.885	0.758
Gaussian scaling	0.803	0.747	0.732	0.701	0.901	0.754
Mixture of all methods	**0.821**	**0.765**	**0.774**	**0.712**	**0.922**	**0.768**

Significant values are given in bold.

The experimental results show that mixing all four methods to augment samples performs better than using a single expansion method. Since all vector-based augmentation methods essentially perturb vector features, we speculate that the mixed methods are more diverse in perturbation, producing hard negative and positive sample vectors that are more effective for model training.

### RQ4: the effectiveness of measures to reduce the risk of overfitting in the CMCS

Since CMCS utilizes a K-means based sampling method to obtain hard negative samples and employs. However, the sampled data may lead to imbalanced categories within the training dataset. The augmentation technique may also result in the model overly relying on the sample features obtained through specific augmentation methods, thereby posing a risk of overfitting. This section explores CMCS's solution to mitigate the potential overfitting caused by sample sampling and augmentation. We also design relevant experiments to verify the effectiveness of the proposed measures.

The K-means based hard negative sample sampling method aims to select samples that are mutually hard negative examples in order to increase the proportion of hard negative samples in the mini-batch, thus enhancing the effectiveness of model training. CMCS adopts three measures to ensure the balance and diversity of samples within the constructed mini-batch, thereby reducing the risk of overfitting that may be caused by the introduction of hard negative samples. First, the sampled samples originate from the real dataset, ensuring the sample features' authenticity. Second, before the training of each new epoch begins, hard negative samples are re-clustered and re-sampled to construct a new mini-batch. Specifically, the encoder updated in the previous epoch is used to re-represent the code samples, followed by re-clustering and re-sampling. Finally, it is ensured that only half of the samples in the constructed mini-batch come from sampling, and the remaining samples are randomly sampled directly from the dataset.

The controllable hardness augmentation method generates positive samples and hard negative samples for contrastive-metric learning. CMCS adopts a fine-grained random augmentation strategy to ensure the diversity of data patterns generated. Specifically, each augmentation in CMCS randomly selects a different augmentation method and the corresponding different amount of perturbation based on four different augmentation methods.

To verify the effectiveness of the above measures in reducing overfitting risk and improving model training performance for CMCS, we conducted experiments on the CodeSearchNet, removing one of the following four measures on CMCS, respectively. The experimental results are shown in Table 6.

A. The augmentation strategy is based on four methods and uses different amounts of perturbation. (After removing A, only one augmentation method is used, and the same amount of perturbation is applied for each augmentation.)
B. The augmentation strategy is based on four methods. (After removing B, only one augmentation method is used, and different amounts of perturbation are applied for each augmentation.)
C. The samples in the minibatch are composed of hard negative samples and randomly sampled negative samples. (After removing C, the minibatch is entirely composed of hard negative samples obtained through sampling.)
D. Before each epoch, re-embedding, clustering, and sampling are performed. (After removing D, only one round embedding and hard negative sample clustering are performed, and random sampling is conducted in each epoch based on the initial clustering results.)

**Table 6:** The performance of different measures to reduce the risk of overfitting.

Language		Ruby	Python	Java	PHP	Go	JavaScript	Average
CMCS	MRR	**0.821**	**0.765**	**0.774**	**0.712**	**0.922**	**0.768**	**0.794**
	NDCG	**0.835**	**0.774**	**0.792**	**0.724**	**0.945**	**0.785**	**0.809**
	SR@1	**0.833**	**0.769**	**0.785**	**0.719**	**0.933**	**0.781**	**0.803**
-A	MRR	0.788	0.716	0.702	0.682	0.885	0.702	0.746
	NDCG	0.798	0.785	0.722	0.699	0.902	0.722	0.771
	SR@1	0.794	0.780	0.715	0.696	0.893	0.715	0.766
-B	MRR	0.803	0.747	0.732	0.701	0.901	0.754	0.773
	NDCG	0.830	0.769	0.752	0.728	0.917	0.771	0.795
	SR@1	0.822	0.761	0.745	0.721	0.912	0.765	0.788
-C	MRR	0.782	0.714	0.756	0.682	0.884	0.623	0.740
	NDCG	0.805	0.739	0.772	0.698	0.912	0.651	0.763
	SR@1	0.793	0.726	0.765	0.688	0.892	0.632	0.749
-D	MRR	0.803	0.735	0.738	0.684	0.902	0.677	0.757
	NDCG	0.823	0.754	0.759	0.698	0.922	0.694	0.775
	SR@1	0.815	0.744	0.746	0.689	0.911	0.682	0.765

Significant values are given in bold.

As can be seen from the results in the second and third rows of Table 6, randomly selecting one of multiple augmentation methods, as well as randomly selecting different perturbations for each augmentation method in the fine-grained random augmentation strategy, can both reduce overfitting and improve model training performance. This proves that the augmentation strategy can generate diverse samples, which is beneficial for balancing the training dataset. Moreover, augmentation techniques increase the quantity and diversity of the original dataset, inherently possessing the property of mitigating overfitting. The results in the fourth row of Table 6 indicate that adding a certain amount of random samples to the mini-batch is beneficial for balancing the types of samples and training the model. The last row of Table 6 demonstrates the benefits of multiple rounds of representation, clustering, and sampling for model training. Multiple rounds of clustering and sampling enhance the randomness and richness of the hard negative samples obtained from sampling in the minibatch and maintain the balance of data in the training minibatch. Moreover, the encoder updated from the previous epoch is used for vector representation before each clustering, enhancing the clustering accuracy. From the above analysis, it can be seen that the measures taken by CMCS can effectively reduce the risk of overfitting and enhance the effectiveness of model training.

### RQ5: determining the optimal parameters for CMCS

#### The choice of the batch size

Batch size refers to the number of samples processed at once during each training iteration. The choice of an appropriate batch size plays a crucial role in the training efficiency and performance of the model. If the batch size is too small, the model may only see a fraction of the data in each iteration, leading to high variance in the learning process. If the batch size is too large, the model sees more data in each iteration, which might require more computational resources and potentially degrade the model's generalization ability. In this section, we explore the optimal batch size to maximize the performance of CMCS. Due to GPU memory limitations, the maximum batch size in this experiment is 128. We conduct experiments on the CodeSearchNet Java dataset, evaluating with five metrics: MRR, NDCG, and SR@1/5/10. The batch sizes tested are 4, 8, 16, 32, 64, and 128, with the results shown in Table 7. The results indicate that a batch size of 32 yields the best code search performance.

**Table 7:** The performance of CMCS with different batch size.

Batch size	MRR	NDCG	SR@1	SR@5	SR@10
4	0.742	0.759	0.747	0.844	0.936
8	0.763	0.780	0.772	0.850	0.945
16	0.770	0.784	0.779	0.856	0.950
32	**0.774**	**0.792**	**0.785**	**0.862**	**0.953**
64	0.753	0.768	0.761	0.845	0.943
128	0.736	0.752	0.739	0.840	0.936

Significant values are given in bold.

#### The choice of the number of clusters *K*

The proposed hard negative sampling method requires K-means clustering on pre-embedded vectors to determine the category of each code snippet in the training set. The K-means algorithm is an unsupervised algorithm based on metric learning, which requires a predetermined number of clusters *K*. We set *K* to to values ranging from 4 to 56, with a step size of 4, to determine the optimal *K* value. We conducted experiments on the CodeSearchNet Java dataset, the results are shown in Table 8.

**Table 8:** The performance of CMCS with different *K* values.

*K*	MRR	NDCG	SR@1	SR@5	SR@10
4	0.721	0.733	0.732	0.522	0.920
8	0.735	0.748	0.742	0.536	0.926
12	0.740	0.753	0.746	0.540	0.928
16	0.753	0.762	0.758	0.545	0.932
20	0.759	0.774	0.768	0.850	0.941
24	0.764	0.780	0.772	0.853	0.945
28	0.770	0.785	0.779	0.858	0.950
32	0.774	0.792	0.785	0.862	0.953
36	0.775	0.794	0.788	0.862	0.592
40	0.777	0.797	0.790	0.863	0.955
44	**0.778**	**0.798**	**0.792**	**0.868**	**0.958**
48	0.774	0.790	0.786	0.861	0.955
52	0.768	0.784	0.780	0.860	0.951
56	0.757	0.775	0.774	0.858	0.948

Significant values are given in bold.

It can be observed from the results that CMCS performs best when *K* is set to 44. We speculate that with more centroids, the code snippets can be divided into more refined categories, resulting in hard negative samples from the same category that are more similar to the original samples, which is beneficial for model training. We also found from Table 8 that the model performance rapidly improved as *K* increased from 4 to 32, but the performance improvement slowed down and reached a plateau as *K* increased from 32 to 44. More clusters mean that the K-means algorithm requires more time to cluster. To balance time and performance, we chose *K* = 32 as the number of clusters for the sampling method.

#### The choice of the proportion of hard negative samples in the batch size

To leverage the beneficial effect of hard negative samples on model training, CMCS samples a certain number of hard negative samples based on K-means, which form a mini-batch combined with randomly sampled general negative samples. Within the mini-batch, too few hard negative samples may prevent the model from being sufficiently trained. At the same time, too many hard negative samples could lead to sample imbalance, potentially causing overfitting and a decline in model performance. This section conducts experiments on the CodeSearchNet Java dataset under 13 different ratios to determine the optimal ratio of the number of hard negative samples to the batch size. Performance is evaluated using five metrics: MRR, NDCG, and SR@1/5/10. As shown in the results of Table 9, when the number of hard negative samples obtained from sampling accounts for half of the batch size, the maximum advantage of the sampled hard negative samples can be exerted.

**Table 9:** The performance of CMCS with the different proportion of hard negative samples in the batch size.

Ratio	MRR	NDCG	SR@1	SR@5	SR@10
0	0.756	0.772	0.765	0.848	0.940
1/16	0.759	0.778	0.770	0.850	0.945
1/12	0.762	0.781	0.770	0.851	0.948
1/8	0.764	0.783	0.774	0.854	0.950
1/4	0.768	0.785	0.778	0.856	0.950
1/3	0.770	0.788	0.780	0.860	0.952
1/2	**0.774**	**0.792**	**0.785**	**0.862**	**0.953**
2/3	0.772	0.790	0.782	**0.862**	**0.953**
3/4	0.767	0.783	0.774	0.855	0.949
7/8	0.764	0.783	0.772	0.852	0.948
11/12	0.760	0.779	0.772	0.851	0.944
15/16	0.756	0.774	0.764	0.846	0.939
1	0.753	0.770	0.759	0.543	0.933

Significant values are given in bold.

#### The choice of the hardness of augmentation

The proposed data augmentation methods are shown in Eqs. (2)–(5), which augments positive and hard negative samples by controlling the hardness parameter. To simplify the description, we summarized multiple perturbation coefficients in the four augmentation methods as a single hardness parameter *θ* as shown in Eq. (6). The hardness values and the corresponding specific values of each perturbation coefficient values are shown in Table 10.

**Table 10:** Correspondence between hardness values and perturbation coefficient values.

*θ*	Linear interpolation		Binary interpolation	Stochastic perturbation	Gaussian scaling
	*α*	*β*	*ρ*	*ρ* (dropout)	*σ*
0.50	0.50	1.50	0.70	0.55	0.50
0.55	0.55	1.45	0.65	0.50	0.45
0.60	0.60	1.40	0.60	0.45	0.40
0.65	0.65	1.35	0.55	0.40	0.35
0.70	0.70	1.30	0.50	0.35	0.30
0.75	0.75	1.25	0.45	0.30	0.25
0.80	0.80	1.20	0.40	0.25	0.20
0.85	0.85	1.15	0.35	0.20	0.15
0.90	0.90	1.10	0.30	0.15	0.10
0.92	0.92	1.08	0.28	0.12	0.08
0.94	0.94	1.06	0.26	0.09	0.06
0.96	0.96	1.04	0.22	0.06	0.04
0.98	0.98	1.02	0.20	0.03	0.02

A larger hardness value indicates that the augmented samples are more similar to the anchor sample and can be used as positive samples. Conversely, a smaller hardness value indicates that the augmented samples are more different from the anchor sample and can be used as negative samples. The hardness value of hard negative samples is between that of positive and negative samples. We augmented positive samples with 8 hardness *θ* values from 0.75 to 0.98, respectively, to test the performance of the bimodal contrastive learning part on java dataset, and the results are shown in Table 11. Then, with the optimal hardness value for positive sample augmentation, we augmented hard negative samples with 8 hardness *θ* values from 0.50 to 0.85, respectively, to test the performance of CMCS on java dataset, and the results are shown in Table 12.

**Table 11:** Impact of augmented positive samples with different hardness on CMCS performance.

*θ*	MRR	NDCG	SR@1	SR@5	SR@10
0.75	0.692	0.711	0.702	0.801	0.911
0.80	0.712	0.733	0.728	0.822	0.932
0.85	0.735	0.752	0.739	0.840	0.941
0.90	0.754	0.768	0.760	0.849	0.945
0.92	0.763	0.777	0.764	0.856	0.948
0.94	**0.774**	**0.792**	**0.785**	**0.862**	**0.953**
0.96	0.750	0.765	0.759	0.840	0.942
0.98	0.704	0.725	0.715	0.815	0.928

Significant values are given in bold.

**Table 12:** Impact of augmented hard negative samples with different hardness on CMCS performance.

*θ*	MRR	NDCG	SR@1	SR@5	SR@10
0.50	0.758	0.770	0.763	0.850	0.943
0.55	0.760	0.775	0.768	0.852	0.946
0.60	0.763	0.779	0.774	0.856	0.948
0.65	0.769	0.785	0.781	0.859	0.951
0.70	**0.774**	**0.792**	**0.785**	**0.862**	**0.953**
0.75	0.767	0.783	0.777	0.858	0.951
0.80	0.764	0.779	0.770	0.856	0.949
0.85	0.760	0.774	0.761	0.853	0.947

Significant values are given in bold.

Tables 11 and 12 show that positive samples generated with a hardness value of 0.94 and hard negative samples with a hardness value of 0.70 have the best effect on CMCS.

#### The choice of the ideal count for augmentation

The proposed augmentation method generates *M1* positive samples and *M2* hard negative samples. The variables *M1* and *M2* also need to be determined for the best performance of CMCS. For simplicity, we set the count for augmentation for each anchor sample to augment *M1* positive and *M2* hard negative samples. In our experiments, we set the minibatch size to 32 accroding to 3.6.1, set *M1* to 1, 3, 5, 7, 9, 11, 13 and 15 to analyze the impact of the augmented positive sample count on CMCS’s performance, set *M2* to 1, 3, 5, 7, 9, 11, 13 and 15 to analyze the impact of the augmented hard negative sample count on CMCS’s performance. We conduct experiments using the Java dataset of CodeSearchNet, and the results are shown in Tables 13 and 14.

**Table 13:** Impact of the *M*_1_ value on CMCS.

*M1*	MRR	NDCG	SR@1	SR@5	SR@10
1	0.724	0.746	0.733	0.836	0.943
3	0.752	0.774	0.763	0.851	0.949
5	**0.774**	**0.792**	**0.785**	**0.862**	**0.953**
7	0.765	0.781	0.775	0.857	0.950
9	0.760	0.774	0.767	0.852	0.948
11	0.757	0.770	0.763	0.849	0.946
13	0.756	0.765	0.760	0.847	0.946
15	0.750	0.757	0.756	0.844	0.944

Significant values are given in bold.

**Table 14:** Impact of the *M*_2_ value on CMCS.

*M2*	MRR	NDCG	SR@1	SR@5	SR@10
1	0.763	0.776	0.771	0.855	0.945
3	0.765	0.780	0.772	0.856	0.947
5	0.768	0.784	0.777	0.858	0.948
7	0.772	0.788	0.780	0.859	0.950
9	**0.774**	**0.792**	0.785	**0.862**	**0.953**
11	0.773	**0.792**	**0.787**	0.861	**0.953**
13	0.765	0.780	0.775	0.856	0.949
15	0.758	0.773	0.764	0.847	0.946

Significant values are given in bold.

The results show that after ten epochs of training, *M*_1_ setting with 5 and *M*_2_ setting with 5 give the best performance for CMCS. The results also indicate that as the number of augmented samples increases, the performance of CMCS exhibits a trend of initially improving and then decreasing. Since sample generation is based on vector-level perturbation, we speculate that generating too many samples may introduce excessive noise and affect the model's ability to learn the features of real samples, resulting in a decrease in performance.

## Related work

### Code search

The initial research on code search was based on information retrieval techniques, and the common practice was to complete the matching between query statements and code by keyword matching methods. Lv et al.^4^ proposed CodeHow which matches keywords between the query and the code snippet API and expands it for code search using a Boolean model. However, the limitation of code search based on information retrieval technology is that it only considers the surface symbolic information of query statements and code snippets, while the deeper semantic content analysis of query statements and code snippets is lacking, which making it difficult to fix the semantic gap between query and code snippet.

Deep learning techniques can capture features from different modalities of data and align them in a vector space, conducting feature analysis based on representation vectors. In image classification research, Tang et al.^38^ proposed a decision fusion module combined with convolutional neural networks, which fully utilizes lower layer features and enhances the completeness of image feature representation, thereby improving image classification accuracy. Research on code search based on deep learning has also significantly improved the performance of code search. CODEnn^8^ constructs two Long Short-Term Memory networks to represent code snippets and queries into vectors separately. Sen et al.^39^ modified CODEnn by using self-attention networks to separately represent code snippets and queries. To fully utilize the various features of the code, Gu et al.^34^ represented the code as an AST, and obtained tree sequences through different traversal and sampling methods. Finally, they obtained code representation vectors by jointly representing the code token sequence with the tree sequence. Guo et al.^33^ used the data flow features of the code to enhance the model's representation of code snippets. Zhang et al.^40^ further parsed the AST of the code into the form of sub-ASTs, integrating more granular syntactic features into the representation of the code. Large code models have superior code pattern recognition and generalization abilities, boasting excellent code search performance. CodeBERT^36^ is a large pre-trained model based on the Transformer architecture that is designed to handle programming languages. It can generalize across various programming tasks and perform excellently. GraphCodeBERT^37^, also based on the Transformer architecture, combines the structural and textual information of code, possessing more robust code feature perception capabilities. Contrastive learning is also used in code search. Syncobert^21^ generates positive samples required for contrastive learning by combining the token sequence of code, AST sequence, and corresponding query sequence in different ways. It aims to learn the complementary information of code semantics and syntax features. HELoC^41^ uses contrastive learning to effectively learn the hierarchical structure of an AST, aiming to more accurately measure the structural similarity between code snippets. Specifically, it uses contrastive learning to enable the network to predict the AST node level and learn the hierarchical relationships between nodes in a self-supervised manner.

Most existing code search research based on contrastive learning only analyzes tokenization and AST features of code, without fully utilizing the multimodal features of code. The CMCS proposed in this paper uses contrastive learning to separately learn the syntax, semantics, data flow, and control flow features of code, possessing stronger capabilities in capturing and representing code features.

### Data augmentation

Data augmentation is a critical technique in contrastive learning, which helps to unsupervised generate positive samples and enables models to learn data features more effectively. Data augmentation for code search can be classified into text-level augmentation and vector-level augmentation.

In text-based augmentation, code augmentation is generally achieved by rewriting the original code samples to augment the code samples, mainly by changing the coding style without altering the code functionality. Rewriting methods include variable renaming, inserting meaningless statements, and changing the order of independency statements^19,25^. Some researchers also represent the same sample multiple times to get positive samples by changing the network parameters^42,43^. For example, Gao et al.^42^ use the Dropout operation to randomly deactivate network nodes and generate positive samples through multiple embeddings. However, text-based expansion methods require the formulation of rewriting rules for preprocessing, and the new samples generated by the augmentation methods also need to be re-embedded, consuming more hardware and time resources. For query augmentation, synonyms, random deletion, and reverse translation are mainly used to achieve expansion^44,45^.

In vector-based augmentation methods, data augmentation is achieved by perturbing the representation vector of the anchor sample. Perturbation is an effective means in various fields^37^. For code search, because this method directly perturbs the represented vectors through mathematical methods, the augmented samples obtained are also in vector form, which is more concise and efficient. Vector-level augmentation techniques are widely used in natural language processing-related tasks^46–48^, using methods such as linear interpolation and random perturbation for augmentation. The proposed CMCS is based on four different perturbation methods, adopting a fine-grained random augmentation strategy. This strategy ensures the diversity of feature patterns in the augmented data, reduces the risk of overfitting, and fully exploits the advantages of contrastive learning.

## Conclusions

In this work, we propose efficient contrastive-metric learning for code search called CMCS, which benefits from positive and hard negative samples. CMCS can generate positive and hard negative samples based on our proposed hardness-controllable sample augmentation method and sample hard negative samples based on the K-means algorithm. We evaluate CMCS on seven advanced code search models and a large-scale dataset. The results demonstrate that CMCS can significantly improve the training efficiency and code search performance. We also conduct extensive experiments to determine the model's most reasonable structure and optimal parameters. In the future, we will explore more effective ways of obtaining high quality positive and hard negative samples to improve training performance.

## Data Availability

The datasets used during the current study available from the corresponding author on reasonable request.
